# A strategy to estimate the rate of recruitment of inflammatory cells during bovine intramammary infection under field management

**DOI:** 10.1186/s12917-017-1078-4

**Published:** 2017-06-08

**Authors:** J. Detilleux

**Affiliations:** Fundamental and Applied Research for Animals and Healh, Sustainable Animal Production, Faculty of Veterinary Medicine, University of Liège, Quartier Vallée 2, Avenue de Cureghem, 6, 4000 Liège, Belgium

**Keywords:** Principal differential analysis, Ordinary differential equation, Cell recruitment rate, Bovine mastitis, Resistance, Tolerance

## Abstract

**Background:**

In most infectious diseases, among which bovine mastitis, promptness of the recruitment of inflammatory cells (mainly neutrophils) in inflamed tissues has been shown to be of prime importance in the resolution of the infection. Although this information should aid in designing efficient control strategies, it has never been quantified in field studies.

**Methods:**

Here, a system of ordinary differential equations is proposed that describes the dynamic process of the inflammatory response to mammary pathogens. The system was tested, by principal differential analysis, on 1947 test-day somatic cell counts collected on 756 infected cows, from 50 days before to 50 days after the diagnosis of clinical mastitis. Cell counts were log-transformed before estimating recruitment rates.

**Results:**

Daily rates of cellular recruitment was estimated at 0.052 (st. err. = 0.005) during health. During disease, an additional cellular rate of recruitment was estimated at 0.004 (st. err. = 0.001) per day and per bacteria. These estimates are in agreement with analogous measurements of in vitro neutrophil functions.

**Conclusions:**

Results suggest the method is adequate to estimate one of the components of innate resistance to mammary pathogens at the individual level and in field studies. Extension of the method to estimate components of innate tolerance and limits of the study are discussed.

**Electronic supplementary material:**

The online version of this article (doi:10.1186/s12917-017-1078-4) contains supplementary material, which is available to authorized users.

## Background

Mastitis remains a major challenge to the dairy industry. Mastitis is characterized by the invasion of the udder by bacteria, their multiplication in the milk-producing tissues, and the production of inflammatory mediators. In response to mediators, inflammatory cells (mainly neutrophils) are recruited from the circulation into the lumen of the alveolus, thus increasing somatic cell counts (SCC) and decreasing the quantity and quality of mastitis milk. Within the gland, neutrophils will phagocyte and destroy invading pathogens. This process is characteristic of the innate immune response to many infectious pathogens.

One control strategy consists of increasing the resistance (host’s ability to reduce parasite establishment) of animals to udder pathogens, either therapeutically or by selection [1]. Mechanisms behind the cow’s ability to resist to mastitis pathogens include traits that reduce pathogen transmission (resistance to infection or “direct” resistance) and pathogen growth rate once infection has occurred (resistance to disease or “indirect” resistance). Different methods have already proven their efficacy in increasing cow’s “direct” resistance, including dry cow antibiotic therapy [2] and vaccination [3]. A contributor to the level of “indirect” resistance is the establishment of a “healthy” immune response in which the infected cow clears the infection and returns to pre-infection status [4]. Among the many components of this healthy immune response, the promptness of the recruitment of blood inflammatory cells in mammary tissues and milk has been shown to be of paramount importance [5–7] but, to the author’s best knowledge, has never been quantified on cows clinically infected under natural conditions.

Ordinary differential equations (ODEs) have been proposed to study the dynamic process of the inflammatory response, including the cell recruitment [8–11]. However, estimation of ODE parameters from complex real data can be difficult, ODEs may not have analytic solutions and numerically solving ODEs can be computationally expensive. Also, stationary confounder effects may not always be included in ODEs and this can be a problem in situations where noise levels are high and the number of data points is low [12].

Here, principal differential analysis (PDA) is proposed to estimate ODE parameters. In this methodology, observed measurements are fitted empirically using spline functions which are then differentiated with respect to time to obtain time-derivative curves. Next, these curves are substituted into the ODEs that can be solved using least-square methods so estimates of the parameters can be obtained [13]. This method has the advantages of being conceptually simpler and more practical than sophisticated numerical methods for solving ODEs by iterative numerical integration. Initials conditions and boundary value need not be known and PDA does not require uniformly sampled data [13]. Missing data can be handled and parameters of interest may be adjusted for known confounders. However, poor splines fit can result in misleading time-derivative information which can lead to poor parameter estimates.

The goal of this study is to test the practicality of PDA in estimating the rate of recruitment of inflammatory cells as an indicator of “indirect” resistance in cows with clinical mastitis.

## Methods

**Fig. 1:**
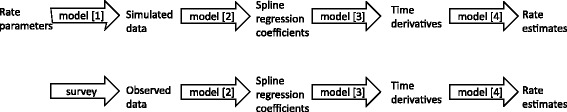
Methodology

To reach this goal, the following procedure was followed (Fig. 1). In the first step, a system of ODEs was proposed to represent the theoretical interaction between inflammatory cells and bacteria during mastitis (model [1]) and to obtain their “model predictions”. In the second step, these predictions (= simulated dataset 1) and SCS collected at specific points in time in cows clinically infected (= observed dataset 2) were smoothed with cubic spline models and variable knots (= models [2]). In step 3, time derivatives of models [2] were computed (= models [3]). Finally, in step 4, rates of recruitment were estimated via a linear regression (= models [4a, 4b]) on these time derivatives. All computations were done on SAS 9.1. To achieve normality of distribution, concentrations in all models were expressed under the log scale as it is done routinely.

### Mathematical simulation of an inflammatory response during mastitis

The model [1] obeyed key biological characteristics observed within an infected udder:

1 $$ \begin{array}{l}\mathrm{dx}/{\mathrm{dt}}_{\mathrm{i}}\kern0.5em =\upgamma\;\mathrm{x}\hbox{-} \upomega \kern0.5em \mathrm{x}\kern0.5em \mathrm{y}\\ {}\mathrm{dy}/{\mathrm{dt}}_{\mathrm{i}}\kern0.5em =\kern0.5em \upupsilon \kern0.5em \left({\mathrm{y}}_0\kern0.5em \hbox{-} \kern0.5em \mathrm{y}\right)\kern0.5em +\upbeta \kern0.5em \mathrm{x}\mathrm{y}\end{array} $$

where x (t_i_) symbolizes the bacterial and y (t_i_) the somatic cell concentrations at a particular point in time (t_i_ = 0, 1, 2, …, 100 time-units; *i* = 1, 2, 3, …, 101). At start, the concentrations are ×_0_ and y_0_. Across time, the bacterial concentration is controlled by the multiplication rate (γ) and the rate at which each inflammatory cell kills bacteria (ω). This last parameter is a first indicator of the level of resistance of the cow. If the animal is insufficiently resistant, ω is low and its inflammatory cells cannot successfully kill bacteria. This is observed in animals with inherited disorders of phagocytic cells [14] and in cows during the peri-parturient period [15]. During health, the concentration of inflammatory cells is controlled by the first term of the second equation where υ is the rate at which cells are recruited and removed in the absence of infection. So, even in the absence of bacteria, there is a standing stock of cells ready to attack. During mastitis, an extra-concentration of activated cells is recruited at a rate β. This is a second indicator of the level of resistance of the host and the indicator of interest in this study. Indeed, an infected host is not very resistant when β is low because activated somatic cells cannot migrate towards the site of infection. This is observed in diseases such as the bovine leukocyte adhesion deficiency syndrome [16].

Initial values were set at ×_0_ = 2 and y_0_ = 10, and a range of values, from 0.001 to 0.50, were set for the different rates in model [1]. Because it is deterministic, the model doesn’t take into account the discreteness of the populations and their random fluctuations so extinction is not possible. Indeed, as the number of cells decreased, the assumption of continuous cell populations is no longer valid and oscillations may occur [17]. As a solution, a threshold of 0.01 was introduced below which the bacterial population is considered extinct, alike in [10].

The system accepts two equilibria, one in the absence and one in the presence of infection. The linear stability of these equilibria was determined by evaluating the characteristic equation (s) of the Jacobian. The equilibrium is stable when all eigenvalues of its Jacobian matrix have negative real parts; it is unstable if at least one eigenvalue has positive real part [18].

### Simulated and observed datasets

Two datasets were used for the second step of the procedure. The simulated dataset (dataset 1) consisted of the values of the concentrations of bacteria (x (t_i_)) and inflammatory cells (y (t_i_)) obtained with [1]. Simulation steps were executed for a period of 100 time-units or until the infection dies out.

The second dataset (dataset 2) came from a survey of 31 commercial dairy farms conducted between January 2008 and December 2011 in the Walloon region of Belgium (Additional file 1)[19]. Herds were enrolled in the regional dairy herds recording system from which test-day somatic cell scores or SCS (i.e., the log-transform of SCC; denoted s (t_i_)) were obtained. Farmers recorded 756 mastitis cases and 1947 SCS values were used in the analyses. Other information included year of calving, parity, days in milk, and number of days between successive events. Clinical mastitis was diagnosed by the breeder when milk from one or more glands was abnormal in color, viscosity, or consistency, with or without accompanying heat, pain, or redness. Only the first mastitis case per parity was considered. Data were collected from 50 days before up to 50 days after the date of mastitis detection. Lactation must include at least two months of lactation. No information was available on bacterial concentrations.

### Cubic Spline models

For the second step of the analysis, records from both datasets were fitted with cubic splines and variable knots:

2 $$ \mathrm{U}\left({\mathrm{t}}_{\mathrm{i}}\right)\kern0.5em =\kern0.5em {\mathrm{g}}_0+{\mathrm{g}}_1\;{\mathrm{t}}_{\mathrm{i}}\kern0.5em +{\mathrm{g}}_2\;{{\mathrm{t}}_{\mathrm{i}}}^2\kern0.5em +\kern0.5em {\mathrm{g}}_3\;{{\mathrm{t}}_{\mathrm{i}}}^3+{\Sigma}_{\mathrm{i}}\;{\mathrm{h}}_{\mathrm{i}}\kern0.5em {\left({\mathrm{t}}_{\mathrm{i}}\kern0.5em \hbox{--} \kern0.5em {\mathrm{k}}_{\mathrm{i}}\right)}^3\kern0.5em {\mathrm{d}}_{\mathrm{i}}\kern0.5em +\kern0.5em \mathrm{e}\kern0.5em \left({\mathrm{t}}_{\mathrm{i}}\right) $$

for *i* = 1, 2, …. 100. The variables U (t_i_) are either x (t_i_) and y (t_i_) (dataset 1) or s (t_i_) (dataset 2) measured at time t_i_ where t_i_ is, for dataset 1, the time since infection (t_i_ = 1, 2,.., 100) and, for dataset 2, the number of days elapsed between the date of milk recording and the date of the case occurrence (t_i_ = −50 to +50 days). Parameters g_0_, g_1_, g_2_, g_3_ and h_i_ are regression coefficients. The knots k_i_ can be any value of t_i_ and the dummy variable d_i_ = 0 if t_i_ ≤ ki and d_i_ = 1 if t_i_ > k_i_. Besides effects in [2], the model for dataset 2 included fixed effects of parity (1, 2, ≥3), days in milk (1, 2 …, 300) and herd-year-season (1, 2 *…,* 174) when the case was observed. These last effects are known factors affecting test-day milk yield and SCS (e.g., [20]). Cubic B-splines are the most frequently chosen spline to fit biological systems because they ally simplicity and biological signification and estimates tend to have high variance when the order of the spline gets larger (e.g., [21]).

Statistically significant knots were selected in a stepwise manner. The final model contained only variables with F-statistics for entry and staying in the model significant at the 0.15 level. The selection stopped at a local minimum of the predicted residual sum of squares (PRESS) criterion. The e (t_i_) were assumed normally and independently distributed with E (e (t_i_)) = 0 and var. (e (t_i_)) = σ_e_^2^. Estimated values of U (t_i_) (= Û (t_i_)) and of regression coefficients (= ĥ and ĝ) were obtained by minimizing the squared differences between U (t_i_) and Û (t_i_). Differences between U (t_i_) and Û (t_i_) and R^2^ values (called R1 values in the following text) were used to evaluate the fit of the model.

### Estimation of rates of recruitment

For both datasets and after stepwise selection, time derivatives (step 3) were computed as:

3 $$ \mathrm{d}\widehat{\mathrm{U}}\kern0.2em \left({\mathrm{t}}_{\mathrm{i}}\right)\kern0.2em {/\mathrm{dt}}_{\mathrm{i}}={\widehat{\mathrm{g}}}_1+2\kern0.5em {\widehat{\mathrm{g}}}_2\kern0.2em {\mathrm{t}}_{\mathrm{i}}\kern0.5em +3\kern0.2em {\widehat{\mathrm{g}}}_3\kern0.2em {{\mathrm{t}}_{\mathrm{i}}}^2+3\kern0.2em {\Sigma}_{\mathrm{p}}\kern0.2em {\widehat{\mathrm{h}}}_{\mathrm{p}}\kern0.2em {\left({\mathrm{t}}_{\mathrm{p}}-{\mathrm{k}}_{\mathrm{p}}\right)}^2\kern0.2em {\mathrm{d}}_{\mathrm{p}} $$

For $\widehat{\mathrm{U}}\kern0.1em \left({\mathrm{t}}_{\mathrm{i}}\right)=\kern0.5em {\mathrm{x}\widehat{\Big(}\mathrm{t}}_{\mathrm{i}}\left),\kern0.5em {\mathrm{y}\widehat{\Big(}\mathrm{t}}_{\mathrm{i}}\right)\kern0.5em \mathrm{and}\kern0.5em {\mathrm{s}\widehat{\Big(}\mathrm{t}}_{\mathrm{i}}\Big)$ with *i* = 1, 2, … 100; p indexes the significant segments (*p* = 1, …. ≤100); ĝ and ĥ are the ordinary least-squares estimates obtained for the regression coefficients in [2]. Differences between dU_i_ (model [1]) and dÛ_i_ (model [3]) were computed.

Finally (step 4), the derivatives were regressed on each system of ODE Eqs. For dataset 1 (model [4a]),

4a $$ \begin{array}{l}\mathrm{d}\widehat{\mathrm{x}}/{\mathrm{dt}}_{\mathrm{i}}=\upgamma \kern0.5em \mathrm{x}\kern0.5em -\upomega \kern0.5em \mathrm{x}\kern0.5em \mathrm{y}\\ {}\mathrm{d}\widehat{\mathrm{y}}/{\mathrm{dt}}_{\mathrm{i}}=\upupsilon \kern0.5em \left({\mathrm{y}}_0\kern0.5em -\mathrm{y}\right)+\upbeta \kern0.5em \mathrm{x}\kern0.5em \mathrm{y}\end{array} $$

and for dataset 2 (model [4b]),

4b $$ \begin{array}{l}\mathrm{d}\mathrm{z}/{\mathrm{dt}}_{\mathrm{i}}\kern0.5em =\kern0.5em \upgamma \kern0.5em \mathrm{z}\kern0.5em -\upomega \kern0.5em \mathrm{z}\mathrm{y}\\ {}\mathrm{d}\widehat{\mathrm{s}}/{\mathrm{dt}}_{\mathrm{i}}\kern0.5em =\kern0.5em \upupsilon \kern0.5em \left({\mathrm{s}}_0\kern0.5em -\mathrm{s}\right)\kern0.5em +\upbeta \kern0.5em \mathrm{z}\;\mathrm{s}.\end{array} $$

The R^2^ values (called R2 values in the following text) were computed to assess the fit of the models in estimating ODE rate parameters. In dataset 2, no information was available on bacterial concentrations necessary to solve model [4b]. As an alternative and to prove the concept of the proposed methodology, they were replaced by z. The values of z were simulated thanks to the first equation of [4b] (dynamics similar to [4a]) with γ = 0.1 and the value of ω that gave the highest R2.

## Results

### Mathematical simulation of an inflammatory response during mastitis

**Fig. 2:**
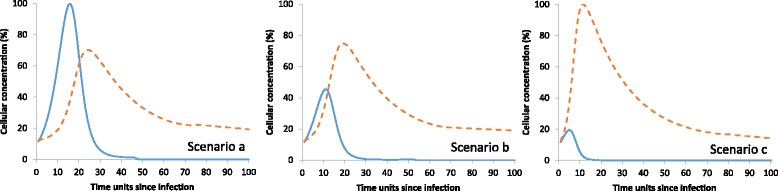
Percentages of the maximum values for y_i_ (dotted line) and x_i_ (plain line) simulated for *i* = 1 to 100 time units since infection and with increasing extra-migration rates when going from scenario ‘a’ to ‘c’

The ODE equations (model [1]) reproduced qualitatively outcomes that can be realistically observed during a healthy response to infection (i.e., scenario ‘a’ in Fig. 2). Such a response is characterized by the following steps. Firstly, bacteria multiply (i.e., x (t_i_) increases) which is followed by an increase in the concentration of inflammatory cells (i.e., y (t_i_) increases). Next, bacteria are killed and their concentrations decrease. At the end of the infection episode, both cell concentrations return to pre-infection values. In scenarios ‘b’ and ‘c’, the increase in x (t_i_) is depressed when compared to scenario ‘a’ because recruitment rates are different (They were set at 0.01, 0.025, and 0.05 cells/time units in scenario a, b, and c, respectively). Peaks for y (t_i_) were at the 24, 19 and 12 time units since infection in scenario ‘a’, ‘b’ and ‘c’, respectively. That is, peaks in cell concentrations occurred faster for higher recruitment rates. Values for the other parameters were unchanged in all three scenarios (i.e., ω = 0.01, γ =0.3, υ =0.05).

The system accepts two equilibria. The first one occurs in the absence of infection for (x_i_; y_i_) = (0; y_0_), i.e. no bacteria and concentration of resident phagocytic cells (y_0_). The equilibrium is stable if ω y_0_ > γ and all eigenvalues of the Jacobian matrix are negative. When ω y_0_ > γ, the rate at which resident cells kill bacteria is greater than their multiplication rate (γ) so bacterial concentration decreases immediately after invasion. When ω y_0_ < γ, it is the reverse: Bacteria multiply and colonize the udder. In the presence of infection, a second equilibrium may occur for (x; y) = (υ/β (1 - (ω y_0_/γ)); γ/ω). At this equilibrium, one of the eigenvalue of the Jacobian matrix is zero and one can’t tell whether the equilibrium point is stable or not.

### Principal differential analysis

In Table 1, one can find some parameter values used to simulate records of dataset 1. Whatever the chosen parameter values, fits of the cubic splines to data (model [2]), and fits of the linear regressions to time derivatives (model [4a]), were excellent. Indeed, R1 and R2 values were above 95% and estimates were close to their corresponding parameter values (see Table 1 for different simulations).

**Table 1:** Model parameters values (E), their estimates (Ê) and standard errors (in parentheses) for 10 different simulations

Simulation		Multiplication rate (□)	Killing rate (□)	Migration rate during health (□)	Extra-migration rate during infection (□)
1	E	0.10	0.01	0.05	0.10
	Ê	0.10 (3.19 10^−4^)	0.01 (2.44 10^−4^)	0.05 (9.66 10^−4^)	0.10 (0.001)
2	E	0.01	0.01	0.01	0.01
	Ê	0.01 (8.21 10^−5^)	0.01 (8.21 10^−5^)	0.01 (8.21 10^−5^)	0.01 (8.21 10^−5^)
3	E	0.10	0.10	0.10	0.10
	Ê	0.10 (1.95 10^−4^)	0.10 (1.95 10^−4^)	0.10 (1.95 10^−4^)	0.10 (1.95 10^−4^)
4	E	0.15	0.15	0.15	0.15
	Ê	0.15 (0.0014)	0.15 (0.0014)	0.15 (0.0014)	0.15 (0.0014)
5	E	0.10	0.10	0.10	0.10
	Ê	0.10 (1.08 10^−4^)	0.10 (1.08 10^−4^)	0.10 (1.08 10^−4^)	0.10 (1.08 10^−4^)
6	E	0.50	0.50	0.50	0.50
	Ê	0.38 (0.026)	0.38 (0.026)	0.38 (0.026)	0.38 (0.026)
7	E	0.10	0.10	0.10	0.10
	Ê	0.101 (2.92 10^−4^)	0.101 (2.92 10^−4^)	0.101 (2.92 10^−4^)	0.101 (2.92 10^−4^)
8	E	0.15	0.15	0.15	0.15
	Ê	0.14 (0.0039)	0.14 (0.0039)	0.14 (0.0039)	0.14 (0.0039)
9	E	0.15	0.15	0.15	0.15
	Ê	0.1467 (0.0014)	0.1467 (0.0014)	0.1467 (0.0014)	0.1467 (0.0014)
10	E	0.15	0.15	0.15	0.15
	Ê	0.149 (5.44 10^−4^)	0.149 (5.44 10^−4^)	0.149 (5.44 10^−4^)	0.149 (5.44 10^−4^)

**Fig. 3:**
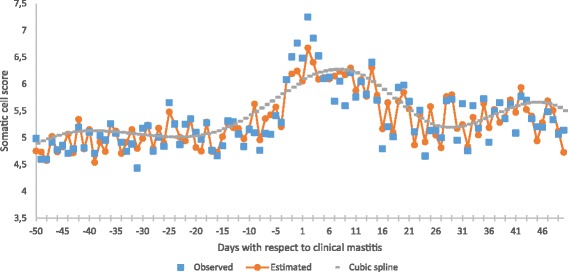
Means of observed and estimated somatic cell scores for 50 days before to 50 days after clinical mastitis. Standard errors are omitted for clarity of the plot

Regarding observed SCS in dataset 2, SCS data were from cows in parity 1 (33%), parity 2 (23%) and parity 3 (44%). Mastitis cases were reported all along the lactation period, with the highest frequencies in the second (11.77%) and third (12.30%) month in lactation. Parity, days in milk and herd-year-season affected significantly SCS. The SCS means were highest in parity ≥3 and in the third month in milk, they decreased across calendar year and were the lowest in winter as compared to summer. Means of observed (s (ti)) and estimated $\left(\mathrm{s}\widehat{\Big(}{\mathrm{t}}_{\mathrm{i}}\Big)\right)$ values and cubic splines are depicted in Fig. 3. The cubic splines are ${\mathrm{s}\widehat{\Big(}\mathrm{t}}_{\mathrm{i}}\Big)$ values adjusted for the effects of parity, days in milk and herd-year-season. Standard errors varied from 0.19 to 0.52 for s (ti) and from 0.16 to 0.41 for ${\mathrm{s}\widehat{\Big(}\mathrm{t}}_{\mathrm{i}}\Big)$. The R1 value was 58.23%. Location of the three knots in the cubic spline was at 14 days before, 12 days after and 27 days after diagnosis of clinical mastitis.

In eq. [(4b)], the value of ω = 0.007 gave the best R2 (39.03%). Estimate of s_0_ is 3.84 (st. err. = 0.70), estimate of υ is 0.0522 (st. err. = 0.50 10^−3^) and estimate of β is 0.0039 (st. err. = 0.112 10^−3^). All are significantly different from null (*p* < 0.05).

## Discussion

Once a cow is infected with mammary pathogens, its immune system mounts a response to them. This response is orchestrated by a hierarchically organized set of molecular, cellular and organismal networks, including the massive influx of inflammatory cells and the killing of bacteria. Although simple, equations in model [1] were able to produce realistic outcomes after intramammary infection [9, 10], as shown in Fig. 2. Using data simulated with model [1], it was verified that PDA (models [2] to [4]) were adequate to estimate parameters of model [1]. Indeed, it fits data almost perfectly as shown in Table 1. This motivates the application of the method to data collected on clinically infected cows (i.e., observed dataset 2). There, the fit was poorer (R1 = 58.23%; R2 = 39.03%). One explanation for this lower fit is that SCS modelled in dataset 2 are only a substitute of the concentrations of phagocytic cells modelled in dataset 1. This last information is often lacking in field studies although, during mastitis, over 90% of the somatic cells are blood neutrophils migrating into the milk [7].

Another explanation is linked to the fact that bacterial concentrations were not observed but simulated using a fixed bacterial growth rate (γ = 0.1). Such information is also often lacking in field studies. Estimates in model [4b] were also not adjusted for the differences that exist between immune responses caused by different bacterial species and strains [22]. Note however that all mastitis cases were suspected to be due to major pathogens, mainly *E. coli* (discussed below). Also, ranking may still be legitimate if we accept that higher killing rates against bacteria that multiply at a rate of 0.1 will also be higher against bacteria that multiply at higher rates.

A third explanation for the poorer fit in observed than simulated data is that ODEs are a simplified version of reality based on various assumptions. For example, it was assumed that a constant proportion of bacterial load was killed by cells at a rate ω, the time needed to process bacteria was negligible and phagocytic cells did not become “satiated”. Another assumption was that concentration of newly migrating cells increased monotonically with concentrations of somatic cells and of bacteria already present in the gland. We may partially accept these assumptions. For example, it was observed in the in vitro study by Li et al. (2004) that rate of bacterial killing of human neutrophils mixed with *S. epidermidis* was only dependent upon the concentration of neutrophils (constant ω). It was also reported that neutrophilic recruitment during mastitis is initiated by inflammatory mediators released from tissue-resident leukocytes when they come into contact with pathogens [6, 23]. This means that a minimum concentration of somatic cells is necessary to initiate the response, which is assumed in the model. A last explanation for the poorer fit in observed than simulated data lies in the cubic spline itself that guarantees continuity and smoothness at the knots at the expense of closeness to data points.

Number and location of knots were estimated from dataset 2: Concentration of somatic cells started to increased 14 days before diagnosis up to the 12 days after diagnosis and returned to values pre-infection values 27 days after diagnosis (Fig. 3). This is characteristic of acute infections with short peaks in SCS, as observed in clinical cases associated with *E. coli* under non-experimental conditions (e. g., [24]). This was also described in quarters experimentally infected with *E. coli*: SCS returned to pre-infection values after a period of 21 to 28 days [25, 26]. An additional argument is that highest SCS were between 6.5 and 7.5 (Fig. 3). Indeed, SCS are regularly higher than 6.4 in infections by major pathogens (as reviewed by [27]).

Even though no information was found in the literature, estimated rates in the absence and presence of bacterial infection (ν and β, respectively) were realistic. The credibility of ν can be discussed in relation to s_0_ that represents SCS in the absence of infection. It was estimated here at 3.84 which is close to the value of 3.91 observed by [28] in cows that were repeatedly and consistently cultured negative. It was slightly below values reported by [29] for bacteriologically negative quarters (between 4.22 and 5.23). The rate of extra-recruitment of cells during infection is represented by β. Its estimate was significantly different from null which is necessary for the resolution of mammary infections [30]. The value of β can be discussed in relation to chemotactic indexes (CI) obtained in in vitro experiments. Indeed, a CI is the number of neutrophils that migrated towards a chemo-attractant to the number of neutrophils that migrated towards a control medium. Similarly, the ratio (βx_i_ + ν)/ν represents SCS that migrated towards mammary gland infected with x_i_ bacteria to SCS that migrated towards uninfected mammary gland. Its value was estimated at 1.3 for x_i_ = 4. It is close to the CI value found by [31] who observed CI of neutrophils from mammary glands inoculated with ~10^4^ CFU/ml of *E. coli* (i. e., x_i_ = 4 on the log scale) was 1.2 times the pre-infection CI value. Similarly, [32] observed the CI of neutrophils from glands infected with *S. aureus* was 1.2 times the CI of non-mastitic (<7.5 10^5^ SCC/ml) mammary secretions. In Fig. 2, bacterial concentration increased as values of β decreased. Correspondingly, [33] observed a delayed chemotactic response in cows with high vs moderate bacterial concentrations during the first 120 h after experimental infection with the same amount of *E.coli*.

Standard errors for υ and β were high and this suggests recruitment rates varied among cows. If confirmed, such finding suggests that recruitment rates could be considered in breeding programs to improve the level of resistance of the population because individual variability is a prerequisite to such programs. Of course, it remains to determine whether this variability is of genetic origin.

As shown in model [1], estimates of direct and indirect resistance levels are ω and ω β, respectively. Estimates of direct and indirect tolerance levels could also be estimated by adding a third equation to model [1]: dm_i_/dt = δ (m_0_ − m_i_) − (η x_i_ + ε y_i_).

Where m_i_ is the milk quantity produced by mammary secretory cells at a particular point in time i. The first term includes the natural rate (δ) at which secretory cells proliferate and die as a result of apoptosis [34]. Parameter η is an indicator of the ability of the cow to tolerate negative effects on milk-secreting tissues (and other components of the mammary gland) of bacterial multiplication and production of toxins, i.e., the ability of the cow to directly tolerate the infection. Parameter ε is an indicator of the ability of the cow to tolerate negative effects of the immune response triggered by the infection and more particularly, milk loss mediated by the increase in the concentration of phagocytic cells, i.e., the ability of the cow to indirectly tolerate the infection. If η = ε = 0, the animal is completely tolerant and produces at the level observed during health. If not, milk drops due to the infection. In this equation, all other effects, such as resource intake, management, month in milk or age, are assumed fixed. It is also assumed that each secretory cell produces the same quality of milk so that m_i_ are directly related to the concentration of secretory cells.

## Conclusions

Results suggest PDA is valuable to estimate, at the individual level and in field studies, rates of neutrophilic recruitment and killing, both of which are components of innate resistance to infectious pathogens. Given the economic and health implications of infectious diseases, a more frequent evaluation of these components in commercial populations could lead to more efficient strategies of disease control and treatments and a better description of host–pathogen interactions.

## Additional file

Additional file 1: No title. Data set used for the statistical analyses with information on the animal identity, parity, month in milk, herd-year-season, somatic cell score and time interval. (XLSX 77 kb)

## Abbreviations

ODE: Ordinary differential eqs.
SCC: somatic cell count
SCS: somatic cell score
